# Application and Utility of Liposomal Neuroprotective Agents and Biomimetic Nanoparticles for the Treatment of Ischemic Stroke

**DOI:** 10.3390/pharmaceutics14020361

**Published:** 2022-02-04

**Authors:** Tatsuya Fukuta, Naoto Oku, Kentaro Kogure

**Affiliations:** 1 Department of Physical Pharmaceutics, School of Pharmaceutical Sciences, Wakayama Medical University, 25-1 Shichiban-cho, Wakayama 640-8156, Japan; 2 Faculty of Pharma-Science, Teikyo University, 2-11-1 Kaga, Itabashi-ku, Tokyo 173-8605, Japan; noku@pharm.teikyo-u.ac.jp; 3 Department of Medical Biochemistry, School of Pharmaceutical Sciences, University of Shizuoka, 52-1 Yada, Suruga-ku, Shizuoka 422-8526, Japan; 4 Department of Pharmaceutical Health Chemistry, Graduate School of Biomedical Sciences, Tokushima University, Shomachi 1, Tokushima 770-8505, Japan; kogure@tokushima-u.ac.jp

**Keywords:** nanoparticles, liposomes, ischemic stroke, cerebral ischemia/reperfusion injury, blood-brain barrier, biomimetic DDS, leukocytes, intermembrane protein transfer

## Abstract

Ischemic stroke is still one of the leading causes of high mortality and severe disability worldwide. Therapeutic options for ischemic stroke and subsequent cerebral ischemia/reperfusion injury remain limited due to challenges associated with drug permeability through the blood-brain barrier (BBB). Neuroprotectant delivery with nanoparticles, including liposomes, offers a promising solution to address this problem, as BBB disruption following ischemic stroke allows nanoparticles to pass through the intercellular gaps between endothelial cells. To ameliorate ischemic brain damage, a number of nanotherapeutics encapsulating neuroprotective agents, as well as surface-modified nanoparticles with specific ligands targeting the injured brain regions, have been developed. Combination therapy with nanoparticles encapsulating neuroprotectants and tissue plasminogen activator (t-PA), a globally approved thrombolytic agent, has been demonstrated to extend the narrow therapeutic time window of t-PA. In addition, the design of biomimetic drug delivery systems (DDS) employing circulating cells (e.g., leukocytes, platelets) with unique properties has recently been investigated to overcome the injured BBB, utilizing these cells’ inherent capability to penetrate the ischemic brain. Herein, we review recent findings on the application and utility of nanoparticle DDS, particularly liposomes, and various approaches to developing biomimetic DDS functionalized with cellular membranes/membrane proteins for the treatment of ischemic stroke.

## 1. Introduction

Ischemic stroke, which accounts for approximately 60% of cerebrovascular disorders, is caused by obstruction of the blood supply into the brain due to thrombi and leads to brain cell damage [1]. Although ischemic stroke is one of the major causes of mortality and severe disability worldwide, therapeutic options remain limited. A thrombolytic agent tissue plasminogen activator (t-PA; alteplase) is the only globally approved therapeutic agent for thrombolytic therapy during the acute phase of restoring cerebral blood flow [2,3]. Unfortunately, due to the risk of cerebral hemorrhage induced by damage to brain endothelial cells, and potential neurotoxic effects resulting from intravenously infused t-PA, the therapeutic time window (TTW; within 4.5 h after onset of an ischemic stroke) is very narrow, which significantly limits the use of t-PA reperfusion therapy in eligible stroke patients [4,5]. Other therapeutic options include endovascular thrombectomy using medical devices, which can be applied to mechanically remove blood clots formed within blood vessels, although this treatment requires specialized skills and cannot be employed to all thrombus sites [6]. In addition, despite successful restoration of blood flow by the abovementioned reperfusion therapies, cerebral ischemia/reperfusion (I/R) injury often occurs and exacerbates patients’ prognoses [7]. Edaravone (Radicut), a radical scavenger, has been used to treat cerebral I/R injury in a few countries (e.g., Japan, China, and India), and was reported to provide certain therapeutic benefits in Europe, though renal disorders following edaravone treatment interfere with its clinical use [8,9]. Therefore, new therapies that can extend the TTW of t-PA and ameliorate cerebral I/R injury are required.

A large number of drug candidates have been developed as potentially effective neuroprotective agents based on findings that indicate that pathological progression of ischemic stroke and subsequent I/R injury is caused by a variety of mechanisms, including inflammation and oxidative stress, among others [10,11]. However, low therapeutic efficacy and adverse side effects caused by insufficient entry into lesioned areas and nonspecific drug distribution have typically resulted in translational failure of those candidate drugs [12,13]. Application of nanoparticle drug delivery systems (DDS) has been of interest as a potential means to address these issues, as they can allow for regulation of the pharmacokinetics of encapsulated drugs, targeting to desired sites by surface modification with functional molecules, and reduction of adverse effects [14]. Herein, we review recent findings on drug delivery using nanoparticles, particularly liposomes, as a therapeutic option for ischemic stroke. We also highlight approaches to developing nanoparticles functionalized with cellular membranes or membrane proteins to mimic the cellular function, which is intended to achieve secure drug delivery to the ischemic stroke region.

## 2. Application of Nanoparticle DDS for the Treatment of Ischemic Stroke

**Figure 1:**
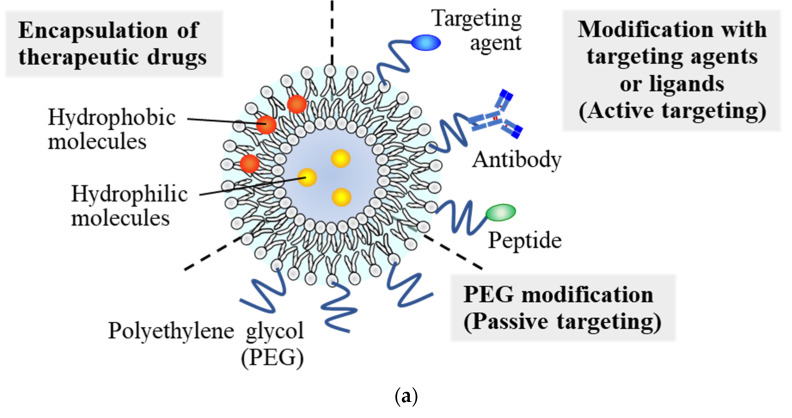

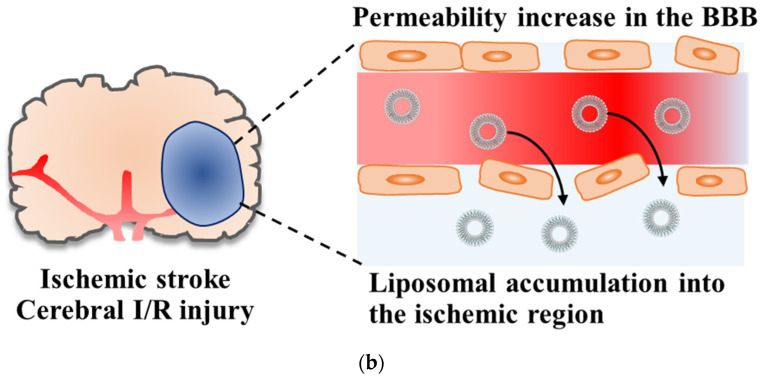
Application of liposome DDS for the treatment of ischemic stroke. (**a**) Schematic diagrams of liposome DDS. (**b**) Accumulation of liposomes in ischemic brain region through the disrupted BBB under conditions of ischemic stroke/cerebral I/R injury.

The blood-brain barrier (BBB) plays a pivotal role in maintaining homeostasis associated with normal functioning of the central nervous system (CNS), and in regulating molecular transport between the blood and the brain. The penetration of drug candidates for the treatment of CNS diseases is typically limited by the BBB, and it is well-known that approximately 100% of macromolecular drugs and >98% of small-molecular drugs cannot cross the BBB under healthy conditions [15]. The BBB also interferes with the transport of systemically administered nanoparticles from the blood into the brain. However, under ischemic stroke conditions, research in animal models and clinical findings indicate that disruption of the BBB occurs around the ischemic area due to several mediators, such as activation of matrix metalloproteinases (MMPs), upregulation of inflammatory cytokines, and reactive oxygen species (ROS) [16,17]. Through the disrupted BBB, blood components, such as water, ions, and immune cells, extravasate into the brain parenchyma, resulting in exacerbation of brain injury [18]. On the other hand, gaps within the disrupted BBB can also be exploited as a route from the bloodstream into the brain tissue for nanoparticle-mediated delivery of therapeutic drugs. In the case of cancer treatment, delivery of anticancer drugs using nanoparticles by traversing the gaps between leaky angiogenic vessels with high permeability is considered a fundamental approach to achieving selective drug delivery into tumor tissues (namely, the enhanced permeability and retention (EPR) effect) [19]. By a mechanism similar to the EPR effect, delivery of neuroprotective agents by nanoparticles into the region of ischemic stroke, as well as the benefits for cerebral I/R therapy, have been demonstrated [20,21]. In this section, we review recent reports on the use of nanoparticle-based DDS for the treatment of ischemic stroke, with a focus on the use of liposomes as drug carriers (Figure 1).

### 2.1. Delivery of Neuroprotective Agents Using Liposomes

Ishii et al. examined the cerebral distribution of fluorescent-labeled polyethylene glycol (PEG)-modified liposomes (PEG-Lip) in transient middle cerebral artery occlusion (t-MCAO) rats prepared by the intraluminal filament method [22]. The liposomes were intravenously injected into t-MCAO rats at various time points after reperfusion following 1-h occlusion, and their distribution was observed at 1 h after liposomal injection. Liposomal accumulation in the ischemic brain hemisphere was clearly observed following administration of the liposomes during the early phases (0–3 h) after reperfusion. Once accumulated, the liposomes remained in the I/R region for 24 h after injection, suggesting that their accumulation in the brain parenchyma could be due to the EPR effect. Moreover, Ishii et al. developed PEG-Lip modified with asialo-erythropoietin (AEPO) [22]. AEPO is known to protect neuronal cells from cerebral I/R injury by exerting anti-apoptotic effects through activation of certain signaling pathways [23]. Modification of AEPO on the surface of PEG-Lip was achieved by post-insertion of distearoylphosphatidylethanolamine (DSPE)-PEG_2000_-AEPO conjugates, which were prepared via a chemical reaction between lysine residues of AEPO and DSPE-PEG_2000_-*N*-hydroxysuccinimide. Liposomalization of AEPO prolongs the blood circulation time and increases accumulation in the I/R region compared with free AEPO. Consequently, intravenous administration of AEPO-modified PEG-Lip immediately after I/R was found to significantly suppress brain cell damage compared with free AEPO at 24 h after I/R [22]. Moreover, another study demonstrated that AEPO modification enabled targeting of PEG-Lip to erythropoietin receptors expressed on the surface of neuronal cells after extravasation from the disrupted BBB. A single injection of AEPO-modified PEG-Lip into t-MCAO rats significantly ameliorated motor functional disorders compared with free AEPO from acute (day 1) to subacute (day 7) phases after I/R [24].

Fukuta et al. prepared PEG-Lip encapsulating the Rho-kinase inhibitor fasudil (Fasudil-Lip) by utilizing an ammonium sulfate gradient between internal (250 mM ammonium sulfate, pH 3.0) and external water phases (phosphate-buffered saline, pH 7.4) [25]. As Rho-kinase activation after an ischemic insult was previously reported to be involved in brain injury via inflammation, oxidative stress, and endothelial cell damage, fasudil is a potential drug candidate for the treatment of ischemic stroke [26]. Intravenous administration of Fasudil-Lip into t-MCAO rats immediately after reperfusion effectively suppressed inflammatory neutrophil infiltration and demonstrated superior neuroprotective effects compared with fasudil solution [25]. In the same report, the authors examined the influence of particle size on liposomal distribution in the ischemic region; results showed that accumulation of PEG-Lip with an average diameter of 100 nm was much greater than the accumulation of PEG-Lip with an average diameter of >200 nm, suggesting that size regulation is an important parameter in developing liposomal neuroprotective agents. In addition to particle size, it is also known that drug release properties from nanoparticles affect therapeutic efficacy [27]. Yanagida et al. reported the impact of the rate of drug release on cerebroprotective effects in cerebral I/R injury using four formulations of Fasudil-Lip by changing the main lipid compositions (distearoylphosphatidylcholine or dipalmitoylphosphatidylcholine) and internal water phases (ammonium citrate or ammonium sulfate). The authors found the therapeutic effects of Fasudil-Lip with a slow drug release rate to be much lower than that with a moderately fast one and suggested that optimization of drug-release properties is likely important to augment the therapeutic outcome of liposomal neuroprotective agents for treating cerebral I/R injury [28].

As post-ischemic inflammation has been reported to be profoundly related to aggravation of cerebral I/R injury, inhibition of inflammatory cascades is a promising approach to improving patient prognoses following ischemic stroke [29]. Since activation of calcineurin results in inflammatory reactions via certain signaling pathways after an ischemic insult, inhibition of calcineurin signaling by an anti-inflammatory agent FK506 (tacrolimus), which has been approved as an immunosuppressant to prevent allograft rejections following organ transplantation, was demonstrated to be effective for the treatment of ischemic stroke [30]. Ishii and Fukuta et al. developed a liposomal formulation of FK506 and demonstrated its utility for the treatment of cerebral I/R injury [31]. FK506 was entrapped in the lipid bilayer membranes of liposomes due to its high hydrophobicity. Compared with FK506 alone, liposomal FK506 showed greater neuroprotective effects through inhibition of immune cell infiltration and anti-apoptotic action and ameliorated cerebral I/R injury [31]. Moreover, the treatment with liposomal FK506 significantly improved motor functional deficits and the cerebral blood perfusion deficit from the acute to subacute phases. The neuroprotective effect of liposomal FK506 was also shown by rgw measurement of mitochondrial activities using positron emission tomography [32]. Liposomes containing cyclosporin A (CsA: immunosuppressant) are another example of a liposomal anti-inflammatory drug with utility that has previously been demonstrated. CsA has been shown to exert neuroprotective effects, especially via inhibition of inflammatory cytokine release (e.g., tumor necrosis factor (TNF)-α, interleukin (IL)-1, 2, etc.) from both immune cells and neurons, but its low bioavailability requires high doses to exert sufficient efficacy [33]. Encapsulation of CsA in nano-sized liposomes allowed for the reduction of the injection dosage of CsA, with remarkable therapeutic effects compared with treatment with the same dosage of free un-encapsulated CsA [34].

With regard to the pathological progression of ischemic stroke, excessively activated *N*-methyl-d-aspartate receptor (NMDAR)-mediated excitotoxicity is recognized as a crucial therapeutic target, as over-influx of calcium ions into neuronal cells via NMDAR stimulated by glutamate represents upstream signaling of oxidative stress and inflammation, among others [35]. To disturb this signaling, Wang et al. developed a transferrin receptor targeting peptide-modified PEG-Lip encapsulating the neuroprotectant ZL006 (5-(3, 5-dichloro-2-hydroxybenzylamino)-2-hydroxybenzoic acid), which selectively inhibits NMDAR activity and prevents glutamate-induced excitotoxicity [36]. Kikuchi et al. reported the use of liposomes encapsulating the NMDAR antagonist ifenprodil, which can be efficiently loaded into liposomes using a pH gradient between internal and external water phases [37]. Both liposomal neuroprotective agents targeting NMDAR signaling exhibited superior therapeutic effects on cerebral I/R injury compared to free drugs in t-MCAO rats.

Liposomes encapsulating the antioxidant baicalin and a liposomal formulation of edaravone have also been evaluated as means of efficiently scavenging ROS produced around the cerebral I/R region [38,39]. Other examples that have been reported of liposomes encapsulating therapeutic drugs with neuroprotective activities against ischemic stroke include liposomal formulations of simvastatin (3-hydroxy-r-methylglutaryl coenzyme A reductase inhibitor) and cytidine-5′-diphosphocholine (citicoline; a vital component of neuronal membranes) [40,41]. Taken together, these findings suggest that the use of liposomes to deliver cerebroprotective agents to the lesioned area offers a promising approach for the treatment of ischemic stroke. Further, drug delivery using liposomes allows for increased delivery efficiency of therapeutic drugs and a reduced injection dosage, which leads to a decrease in the risk of potential adverse side effects for future clinical applications. The use of liposomes as drug carriers also has the advantage that various pharmaceutical modalities, from hydrophobic/hydrophilic small molecule drugs to macromolecular drugs (e.g., proteins, nucleic acid therapeutics), can be applied for the treatment of ischemic stroke. Table 1 shows representative examples of liposomal neuroprotective agents reviewed in this section.

### 2.2. Ligand-Mediated Targeting with Liposome DDS

Modification of liposomal surfaces with certain ligands, such as peptides and antibodies, allows for targeted drug delivery to cells expressing specific molecules, resulting in an increase in the therapeutic efficacy of encapsulated drugs. The active targeting strategy using ligand-modified liposomes is a common approach and has been applied for the treatment of various diseases, including cancer and inflammatory diseases. In this section, we review several examples of ligand-mediated active targeting strategies using liposomes to treat ischemic stroke.

Agulla et al. identified that heat shock protein 72 (HSP72) is selectively upregulated in the peri-infarct region following an ischemic insult and becomes an appropriate molecular biomarker for ischemic stroke [43]. The authors designed anti-HSP72 antibody-modified targeting liposomes and tested their usefulness both in vitro and in vivo. The HSP72 antibody-modified liposomes were selectively associated with HSP72-expressing primary astrocytes and demonstrated superior targeting ability to the lesioned area in t-MCAO rats in comparison to PEG-Lip. Results also demonstrated that intravenous administration of HSP72 antibody-modified liposomes encapsulating the neuroprotective agent citicoline exerted significantly greater protective effects than groups administered either citicoline or PEG-Lip encapsulating citicoline [43].

Transferrin receptor (TfR) is highly expressed on cerebral endothelial cells and can transport transferrin-bound iron atoms across the BBB via transcytosis, which makes TfR a promising target for drug delivery to the brain [44,45]. Nanoparticles modified with the TfR-targeting peptide HAIYPRH (T7) were previously reported to achieve selective targeting of brain tumors across the BBB [46]. Wang et al. reported the utility of T7-modified liposomes loaded with ZL006 for ischemic stroke therapy [36]. Modification of the liposomal surface with T7 peptide significantly increased the ZL006 drug concentration in the brains of healthy mice, and also increased the number of liposomes (injected at the time of reperfusion following 2-h ischemia) that accumulated in the ischemic brain at 6 and 24 h after the start of ischemia. Accordingly, treatment with T7-modified ZL006 liposomes significantly reduced the damaged brain volume compared with non-targeted liposomes, similar to the results described above for HSP72-targeting immunoliposomes [43].

To identify a novel targeting peptide for the region of ischemic stroke, Hong et al. performed an in vivo phage display using t-MCAO rats [47]. Through three cycles of in vivo screening, the authors identified a candidate peptide, namely CLEVSRKNC, termed stroke-homing peptide (SHp). Although the target molecules of SHp have not been identified, SHp exhibits the ability to home in specifically on the region of ischemic stroke and was shown to detect apoptotic neuronal cells after I/R. By employing SHp, Zhao et al. developed dual-targeting liposomes modified with SHp and a TfR targeting T7 peptide [42]. This dual-targeting strategy aimed to transport the liposomes across the injured BBB through TfR-mediated transcytosis and subsequently target neuronal cells in the ischemic region. In vitro experiments using rat brain capillary endothelial cells indicated that modification of liposomes with T7 peptides facilitated efficient transport across the endothelial cell layer, whereas liposomal modification with SHp increased binding to glutamate-stimulated PC-12 cells. Moreover, significantly greater amounts of intravenously injected liposomes accumulated in the ischemic region of MCAO rats on modification with both peptides compared with liposomes modified with each peptide alone. Consequently, dual-targeting liposomes encapsulating ZL006 showed remarkable neuroprotective effects by enhancing local drug concentrations in the brain while reducing their adverse effects [42]. The abovementioned findings demonstrate that ligand-mediated active targeting liposomes can strongly interact with specific molecules highly expressed in the ischemic region and thus could improve the delivery efficiency and therapeutic efficacy of cerebroprotective agents.

On the other hand, Ishii et al. demonstrated that the neuroprotectant AEPO can be applied not only as a therapeutic agent but also as a targeting ligand to the cerebral I/R region [24]. It was previously reported that EPO receptor expression on neurons is upregulated by hypoxia-inducible factor 1α after an ischemic insult [48]. In addition to neuronal cells, the level of EPO receptor expression is known to increase in vascular endothelial cells during cerebral ischemia [49]. Indeed, accumulation of fluorescently-labeled AEPO-modified PEG-Lip was more densely observed than non-modified PEG-Lip around neuronal cells in the cerebral I/R region. In addition, AEPO-modified PEG-Lip was shown to accumulate in the I/R region, not only via extravasation from the disrupted BBB but also by binding to injured cerebral vessels, while their accumulation in cerebral vessels was hardly observed in the non-ischemic hemisphere. Hence, modification of nanoparticles, including liposomes, with AEPO may be useful to develop targeting DDS with therapeutic potency against ischemic stroke. Encapsulation of therapeutic agents with other mechanisms of action in AEPO-modified liposomes offers an interesting approach to designing highly functional DDS with targeting abilities and synergistic therapeutic mechanisms.

Cyclic RGD (Arg-Gly-Asp-_D_-Tyr-Lys) peptides are well-known as active targeting ligands for inflammation-related diseases [50]. In particular, the cRGD motif exhibits binding affinity for αv integrins, such as αvβ1 and αvβ3, which are expressed on cancer cells and inflamed angiogenic endothelial cells. Studies have demonstrated the applicability of liposomes decorated with cRGD peptides on the liposomal surface as a DDS for ligand-mediated targeting in cancer therapy [51,52]. Additionally, as cRGD can specifically bind to glycoprotein IIb-IIIa integrin expressed on activated platelets, cRGD modification of liposomes enables thrombus-targeting for the delivery of t-PA in the treatment of stroke [53]. However, with regard to cRGD-mediated accumulation of liposomes in tumor tissue, there is the possibility that cRGD-modified liposomes bind to and are taken up by integrin αvβ3-expressing phagocytes; the phagocytes may then actively transport phagocytosed liposomes to tumor tissues, resulting in cRGD-mediated active targeting to the tumor site [54]. Sofias et al. suggested that cRGD-mediated active liposomal accumulation in the tumor is multifaceted, and that “nanoparticle hitchhiking” by phagocytes could considerably contribute to the accumulation of cRGD-decorated liposomes in deeper regions of the tumor [54]. By employing a similar strategy, Hou et al. demonstrated the utility of leukocyte-mediated delivery of cRGD-modified liposomes into the region of the brain associated with ischemic stroke [39]. As a series of inflammatory responses following cerebral ischemia leads to recruitment of leukocytes to the ischemic core and penumbra region, leukocytes were applied as “Trojan horses” for targeted delivery of cRGD-modified liposomes into the lesioned sites. The efficient uptake of cRGD-modified liposomes was observed in neutrophils/monocytes of cerebral I/R model rats, resulting in leukocyte-mediated transport of the liposomes to ischemic neurons. Similarly, the antioxidant edaravone encapsulated in cRGD-modified liposomes showed superior neuroprotective effects compared to non-modified liposomes. In addition to ischemic stroke, neuroinflammation induced by leukocyte recruitment is involved in the pathological progression of a number of central nervous system disorders, such as Alzheimer’s disease, glioma, and multiple sclerosis [55]. Hence, liposomal delivery of therapeutic agents using leukocytes or other blood-circulating immune cells offers a promising means of ligand-mediated active targeting to inflammation sites (including the ischemic stroke region). Table 2 shows representative examples of targeting ligands that have been employed for surface modification and that are reviewed in Section 2.2 and Section 2.3. 

### 2.3. Application of Other Nanoparticle DDS

As described above, nano-sized liposome-mediated delivery of neuroprotectants offers a hopeful avenue for the development of new therapies for the treatment of ischemic stroke. In addition to liposomes, various research efforts have focused on the design of synthetic nanoparticle-based DDS. In this section, we review some examples of the synthetic nanoparticle systems developed for the delivery of neuroprotective agents.

Poly(lactic-co-glycolic) acid (PLGA) is a Food and Drug Administration (FDA)-approved synthetic polymer with favorable biocompatibility and biodegradability properties and has been widely used for medical applications, including the treatment of ischemic stroke. Reddy and Labhasetwar reported PLGA-nanoparticles encapsulating the potent antioxidative enzyme superoxide dismutase (SOD) to prevent oxidative stress from being induced following an ischemic stroke [59]. The PLGA-nanoparticles were injected into t-MCAO rats via the intracarotid artery (ICA) to increase the delivery efficiency to the brain compared with the intravenous route. Treatment with PLGA-nanoparticles encapsulating SOD showed a 65% reduction in the volume of brain damage, while the same dosage of SOD solution alone resulted in only a 25% reduction compared with the control group, suggesting that the use of PLGA-nanoparticles enables sustained release of SOD into the lesioned area. Consequently, the nanoparticles suppressed brain edema by preventing ROS-related BBB disruption and protected neuronal cells from apoptosis [59]. In another report, Petro et al. developed PLGA-nanoparticles co-encapsulating two antioxidative enzymes, namely SOD and catalase (CAT), and investigated the neuroprotective effects of the system. Although a comparison between PLGA-nanoparticles co-encapsulating SOD/CAT and those encapsulating each enzyme alone was not performed, co-delivery of SOD/CAT via the ICA route showed remarkable protective effects via multiple mechanisms, including antioxidative and anti-inflammatory effects, which resulted in the promotion of endogenous neurogenesis around the injured brain region [60].

Han et al. developed PLGA-nanoparticles modified with a targeting peptide against MMP-2, namely chlorotoxin (CTX) [57]; upregulation of MMP-2 expression has been reported around the ischemic stroke region [61]. Lexiscan, a small molecular drug approved by the FDA as an imaging agent for myocardial perfusion, was also encapsulated in PLGA-nanoparticles as a BBB modulator, as lexiscan had previously been demonstrated to induce a transient increase in BBB permeability via activating adenosine A2a receptor signaling, resulting in the promotion of drug delivery to the brain [58]. Owing to the surface modification with CTX and the pharmacological effect of lexiscan, accumulation of the intravenously injected PLGA-nanoparticles in the ischemic region of t-MCAO mice was significantly increased compared with the accumulation of plain PLGA-nanoparticles. Moreover, a therapeutic peptide, NEP1-40 (Nogo-66 receptor antagonist), co-entrapped in the CTX-modified PLGA-nanoparticles together with lexiscan, significantly ameliorated ischemic brain injury compared with a simple injection of free NEP1-40 [58].

A transient BBB opening strategy via adenosine A2a receptor signaling was also reported using poly(amidoamine) (PAMAM)-dendrimer as a drug carrier. The PAMAM-based adenosine A2a receptor agonist, termed a nanoagonist, was developed via the chemical conjugation of a purine nucleotide derivative with a high binding affinity to the receptor [56]. Surface modification of the nanoagonist with cRGD enabled selective targeting to the inflamed cerebral vessels around the site of ischemic stroke. The cRGD-modified nanoagonist could transiently open the injured BBB and increase the delivery efficiency of subsequently administered SOD into the ischemic brain, resulting in the exertion of efficient antioxidative effects [56].

As mentioned above, the application of synthetic nanoparticles for the design of therapeutic systems is suggested to offer a promising treatment approach for ischemic stroke. While we reviewed only a few examples of the use of PLGA-nanoparticles and PAMAM dendrimers, several review articles have also highlighted the utility of a variety of other synthetic nanoparticle-based DDS [21,62,63].

## 3. Combination Therapy with Thrombolytic Agents and Nanoparticulate Neuroprotective Drugs

As highlighted above, neuroprotection via delivery of neuroprotective agents using nanoparticle-based DDS offers a promising approach to prevent the expansion of secondary brain injury. In addition to neuroprotection, appropriate removal of blood clots by thrombolysis with t-PA, a standard treatment worldwide, is also indispensable for ischemic stroke therapy, despite the fact that the proportion of applicable patients remains very low [4]. The main reasons for the limited eligibility are the narrow TTW of t-PA injection and safety concerns, such as the risk of cerebral hemorrhagic transformation and harmful effects induced by t-PA-activated neurotoxic signal pathways [64]. Thus, therapeutic strategies that can alleviate the adverse effects of t-PA and improve its effectiveness by prolongation of the TTW are needed. In this section, we review research efforts aimed at increasing the utility of t-PA therapy.

It has been reported that the integrity of the BBB can become compromised during the acute phase of brain ischemia, as well as after reperfusion [65]. This phenomenon has been suggested to enable nanomedicines to enter the ischemic region prior to reperfusion (i.e., under ischemic conditions) to provide protection before the start of t-PA therapy. In permanent MCAO (p-MCAO) rats prepared by the intraluminal filament method, accumulation of fluorescently- and radio-labeled liposomes in the ischemic region was demonstrated using ex vivo fluorescence imaging and positron emission tomography, respectively [66,67]. Moreover, intravenous administration of liposomal FK506 into t-MCAO rats 1 h before reperfusion effectively suppressed oxidative damage and expansion of I/R injury, suggesting the potential of nanoparticle-mediated neuroprotectant delivery prior to reperfusion [68]. MCAO rats prepared by photochemically-induced thrombosis (PIT) have been beneficial in demonstrating the utility of combination therapy with neuroprotective agents and t-PA, as a blood clot can be artificially formed in the MCA and subsequently deleted by t-PA administration, resulting in restoration of blood flow [69]. These properties allowed for the evaluation of the therapeutic efficacies of both thrombolytic agents and drug candidates, as well as combination effects [70]. By employing MCAO rats prepared using the PIT method, Fukuta et al. reported the utility of combination therapy using liposomal neuroprotectants and t-PA for the treatment of ischemic stroke [71]. Intravenous administration of Fasudil-Lip prior to t-PA injection markedly suppressed t-PA-induced BBB damage and also inhibited activation of MMP-2 and -9, which are enzymes that are greatly associated with cerebral hemorrhagic transformation following thrombolytic therapy [61]. The combination treatment with Fasudil-Lip plus t-PA showed significantly greater cerebroprotective effects compared with each treatment alone by inhibition of neutrophil infiltration into the lesioned area, which resulted in an extension of the TTW of t-PA in MCAO rats [71]. With regard to combination therapy using t-PA and neuroprotectants, it is possible that reperfusion via thrombolytic agents may promote entry of the therapeutic agents into the ischemic area [72]. Fukuta et al. investigated the impact of t-PA administration on the accumulation of liposomes, and demonstrated that accumulation in the lesioned area was significantly increased by t-PA as it promoted extravasation of the liposomes from cerebral vessels, suggesting that the combined effects of Fasudil-Lip and t-PA are synergistic and result from promotion of liposomal accumulation in the brain parenchyma [73]. These findings suggest that the combination of liposomal neuroprotective agents should increase the efficacy of t-PA thrombolytic therapy by prolonging t-PA’s TTW and reducing the risk of a cerebral hemorrhage.

The short half-life (<5 min) of t-PA in the blood has also been recognized as another issue limiting its application, as enzymatic degradation and rapid clearance of t-PA are known to decrease its therapeutic potency [74]. To prolong the circulation of t-PA in the blood, Kim et al. entrapped t-PA in PEG-Lip [75]. Tang et al. developed gold nanoparticles with their surfaces chemically conjugated with t-PA [76]. Both efforts were shown to prolong the t-PA half-life by avoiding enzymatic degradation; however, such nanoparticles cannot protect brain cells from secondary I/R injury. To simultaneously prolong t-PA’s blood-circulation time and ameliorate cerebral I/R injury, Mei et al. developed self-assembled antioxidative nanoparticles encapsulating t-PA [77]. To suppress I/R-induced oxidative stress, the nanoparticles were prepared using strong antioxidant 4-amino-2,2,6,6,-tetramethylpiperidine-1-oxyl (4-amino TEMPO) moieties containing polymers. The 4-amino-TEMPO-conjugated redox nanoparticles encapsulating t-PA, termed t-PA@RNP, were found to prevent brain damage and lessen neurological deficits via effective suppression of I/R-induced oxidative stress in MCAO mice prepared by the PIT method. In addition, t-PA@RNP markedly extended the half-life of t-PA, with enzymatic activities in the systemic circulation (pH 7.4). Moreover, to capitalize on the decrease in pH around the ischemic region due to acidosis [78], t-PA@RNP was designed to release t-PA from the nanoparticles by dissociation under acidic conditions, thus allowing for efficient thrombolysis by the released t-PA [77].

With regard to combination therapy with t-PA and nanoparticle neuroprotective agents, the influence of t-PA on the physicochemical properties of nanoparticles must be considered since their properties are greatly related to the therapeutic efficacy of nanoparticle drugs (as described in Section 4). In the abovementioned study to develop liposomes encapsulating t-PA, the particle size, polydispersity index, and ζ-potential of the liposomes were hardly changed regardless of t-PA encapsulation [75]. In the studies using gold nanoparticles and t-PA@RNP, their particle sizes were slightly increased (<10–15 nm) by t-PA conjugation [76,77]. However, the thrombolytic activity of t-PA in vivo was found to increase due to the prolongation of the half-life of t-PA as a result of preventing its enzymatic degradation. In the case of combined treatment with t-PA and Fasudil-Lip, the t-PA treatment significantly increased the accumulated liposomes in the ischemic region [73]. Accordingly, the therapeutic effect of Fasudil-Lip was synergistically found to be increased compared to monotherapy with Fasudil-Lip or t-PA. These findings suggest that the combination therapy with t-PA could not have a significant negative impact on the physicochemical properties and therapeutic efficacy of nanoparticle drugs.

**Figure 2:**
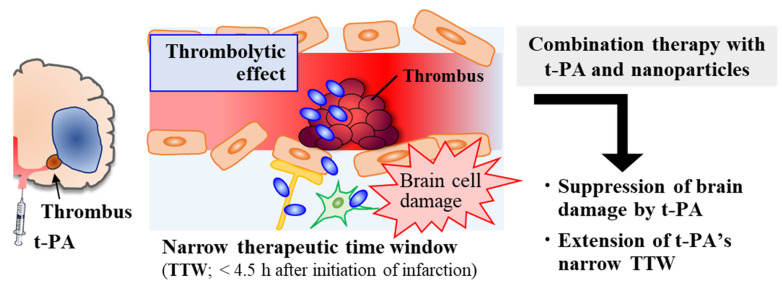
Combination therapy with the thrombolytic agent t-PA and neuroprotective nanoparticles.

For clinical ischemic stroke therapy, improvement of the efficiency of t-PA thrombolytic therapy and prevention of cerebral I/R injury are both desirable aims [79]. The above findings suggest that combination treatment with nanomedicines and t-PA offers the potential to prolong the narrow TTW of t-PA by decreasing the risk of cerebral hemorrhage and preventing neurotoxic events (e.g., MMP activation) induced by t-PA. Moreover, the accumulated nanomedicines in the lesioned area can suppress the pathological progression of cerebral I/R injury. Combination therapy may improve therapeutic outcomes for patients eligible for thrombolytic therapy (Figure 2). Table 3 summarizes the use of nanoparticles for delivering t-PA and the combination therapy options reviewed in this section.

## 4. Factors That Affect Therapeutic Efficacy of Nanoparticles Encapsulating Cerebroprotective Agents

The usefulness of nanoparticle DDS, particularly liposomes, for the treatment of ischemic stroke is highlighted above. To increase the therapeutic efficacy of the nanoparticle drugs on ischemic stroke, their physicochemical properties (e.g., particle size, surface charge) should be considered, as those properties are greatly related to biodistribution, stability in the bloodstream, and the therapeutic efficacy of nanoparticles encapsulating cerebroprotective agents. In this section, we discuss certain factors that affect the therapeutic efficacy of nanoparticle drugs for the future development of new therapeutic agents against ischemic stroke.

The particle size is a crucial factor to determine the fate of systemically administered nanoparticles for ischemic brain targeting. Based on the investigations using t-MCAO rats, Fukuta et al. demonstrated that PEG-Lip with approximately 100 nm in diameter showed wider accumulation in the ischemic region compared with those of over 200 nm [25]. On the other hand, no accumulation of liposomes with over 800 nm in diameter was observed, suggesting that the gaps in the BBB around the ischemic region could be large enough to allow for permeation of liposomes smaller than 200 nm. By using PLGA nanoparticles with different sizes (100, 200, and 800 nm), Cruz et al. also reported similar results in traumatic brain injury model mice [80]. In the case of local administration of PLGA nanoparticles in the brain, Zhou et al. showed that nanoparticles with a diameter of less than 100 nm (70 nm) can diffuse deeply in the brain parenchyma, while larger nanoparticles (147 nm) lack diffusivity in the brain due to their dense tissue [81]. Although systematic investigations on the accumulation of intravenously injected liposomes of less than 100 nm remain unreported, it is speculated that therapeutic agents could be delivered over a wider region in a brain with ischemic stroke by employing liposomes of less than 100 nm when considering the liposomal diffusivity. In recent years, the microfluidic method has attracted attention for how to easily prepare stable and size-tuned liposomes with less than 100 nm in diameter [82]. Hence, by employing the microfluidic method, a systematic examination of the particle size of liposomes can be carried out in future research to increase the accumulation and diffusivity of liposomes in the ischemic stroke region and enhance the therapeutic efficacy of encapsulated neuroprotective agents.

The surface charge also influences the biodistribution of systemically administered nanoparticles. As the luminal side of the BBB is negatively charged owing to proteoglycans expressed on endothelial cells, positively charged nanoparticles can use adsorptive-mediated transcytosis to cross the BBB [83]. However, cationic nanoparticles (>+10 mV) induce serum protein aggregation and display high macrophage uptake and clearance by the mononuclear phagocyte system (MPS), which results in a low residence time in cerebral vessels and limited delivery of nanoparticles to the brain [84]. It is also known that cationic nanoparticles aggregated by electrostatic interaction with blood cells tend to be trapped in small capillaries in the lungs. Moreover, cationic nanoparticles pose a risk of inducing greater toxicity than anionic and neutral nanoparticles. With regard to the ability of anionic and neutral nanoparticles to circulate in the blood, Levchenko et al. demonstrated using liposomes that the blood clearance rate of anionic liposomes (<−40 mV) is significantly faster than that of PEG-modified neutral liposomes (from −10 to +10 mV) [85]. The authors also showed that anionic liposomes exhibit a higher rate of MPS uptake in the liver than PEG-modified neutral liposomes, implying that phagocytic cells prefer to engulf anionic liposomes compared to PEG-modified neutral ones [85]. With regard to the treatment of ischemic stroke, PEG-modified neutral liposomes with a long blood circulation period work advantageously for accumulation in the brain parenchyma by passing through the disrupted BBB via the EPR effect, resulting in an increase in the therapeutic efficacy of drugs delivered by liposomes. In addition, a study using polystyrene nanoparticles and PLGA nanoparticles showed that coating those nanoparticles (40 and 100 nm) with a high density of PEG brings about a significantly greater increase in diffusion through the brain extracellular spaces compared to the non-PEG-coated nanoparticle in the case of those intracranially injected into mice [86]. Collectively, PEG modification of the nanoparticle surface should be important to increase the drug delivery efficiency and therapeutic efficacy of nanoparticle drugs for ischemic stroke therapy by improving both the blood circulation and diffusivity in the brain parenchyma. 

In addition to their charge, the aggregation behavior of nanoparticles is an important factor to determine the biodistribution of systemically administered nanoparticles, as aggregation-induced increases in the particle size accelerate their trapping by the reticuloendothelial system (RES), resulting in a decrease of therapeutic efficacy. Thus, suppression of nanoparticle aggregation in the blood contributes to prolonging the blood circulation period of nanoparticles, which allows for an increase in their therapeutic efficacy. For example, the abovementioned PEG modification has been reported to be useful for preventing nanoparticle aggregation by serum proteins [87,88]. Furthermore, the application of nanoparticles, particularly liposomes, can protect encapsulated drugs, such as small molecular hydrophilic/hydrophobic drugs, peptides, and proteins (e.g., t-PA and SOD), against enzymatic metabolism and degradation, resulting in enhancement of their therapeutic efficacy in target sites. However, regarding the liposomal and other nanoparticle preparations for ischemic stroke therapy described in this review, detailed investigations of aggregation behavior, temperature-stability, and enzyme-stability after long-term storage, and the influence of storage conditions on those parameters and their therapeutic efficacy, have hardly been performed. To develop nanoparticle-based therapeutic agents for ischemic stroke, further examinations on pharmaceutical viewpoints, such as temperature/enzyme-stability and storage conditions, and their relation to the therapeutic effects of nanoparticles on ischemic stroke, will be required.

As described in Section 2.2 and Section 2.3, surface modification of nanoparticles with targeting ligands improves the delivery efficiency and therapeutic efficacy of encapsulated cerebroprotective agents. When modifying nanoparticles with targeting ligands, phospholipid- or polymer-PEG conjugates with reactive functional groups, such as *N*-hydroxysuccinimide and maleimide, are generally employed. These conjugates can easily be reacted with targeting ligands via reactive functional groups and can subsequently be incorporated on nanoparticles. Importantly, in the case of preparing nanoparticles incorporating targeting ligands, optimum modification amounts of ligands should be considered. Anraku et al. previously designed glucose-integrated polymeric micelles for brain targeting and demonstrated the presence of optimal glucose modification amounts to exert their desired function [89]. On the other hand, depending on the kind of ligand, excessive modification impairs the stealth function of PEG and leads to increased recognition of the nanoparticles as foreign substances by the RES [90]. Therefore, for future research to develop targeting ligand-modified nanoparticles for ischemic stroke therapy, a detailed investigation is required into the effect of incorporating each ligand on the biophysical properties of nanoparticles, to optimize the functionality of resultant formulations.

## 5. Recent Approaches to Ischemic Stroke Therapy Using Biomimetic DDS

Nanoparticle-mediated delivery of cerebroprotective agents through the disrupted BBB has been recognized as a useful strategy for ischemic stroke treatment. On the other hand, it has been previously reported that pericyte contraction elicited by oxidative-nitrative stress causes cerebral microcirculation dysfunction, which disturbs drug entry into the I/R region via the blood circulation despite successful reperfusion [91]. Following ischemic stroke, the I/R-induced increase in the permeability of the BBB has been shown to exhibit time-dependent and biphasic changes, whereby the increase in vascular permeability after I/R peaks once at 3–6 h (acute phase), with delayed BBB opening occurring at 48–72 h (subacute phase) [61,92]. These phenomena limit the entry of nanoparticles into the I/R region, resulting in poor accumulation (<1% of injected dose). Indeed, Ishii et al. found that accumulation of liposomes in the I/R region was markedly decreased when fluorescently labeled PEG-Lip was administered at 6 h following reperfusion to t-MCAO rats, compared with PEG-Lip injected prior to 6 h following reperfusion [22]. Moreover, liposomal accumulation was hardly detected in the case of injection at 24 h after reperfusion. Al-Ahmady et al. also reported similar results. In particular, the authors found that PEG-Lip injected at an early time point (2.5 h after reperfusion) can efficiently accumulate in the I/R region compared with PEG-Lip injected between 4.5 and 24 h after the start of reperfusion. The authors suggested the possibility that the delayed BBB disruption (during 48–72 h after I/R) allows for liposomal entry into the I/R region [93]. As described in Section 2 and Section 3, ligand-mediated targeted nanoparticle delivery is one approach to increasing the delivery efficiency into the I/R region. However, it should be noted that although modification of the nanoparticle surface with targeting ligands enables an increase in affinity to certain cells expressing target molecules, marked enhancements of nanoparticle extravasation from blood vessels or entry into the tissue parenchyma cannot generally be obtained [27,94]. Hence, the above limitations associated with nanoparticle delivery have to be overcome to securely deliver drugs into the ischemic stroke region.

To this end, recent efforts have focused on the unique properties of circulatory cells (e.g., erythrocytes, leukocytes, platelets, etc.) to construct nanoparticles mimicking their properties, termed “biomimetic DDS” [95]. In the following section, we highlight the application of biomimetic DDS for ischemic stroke therapy, as well as our recent efforts to prepare biomimetic DDS using a convenient intermembrane protein transfer method.

### 5.1. Biomimetic DDS Prepared with Circulatory Cells

**Figure 3:**
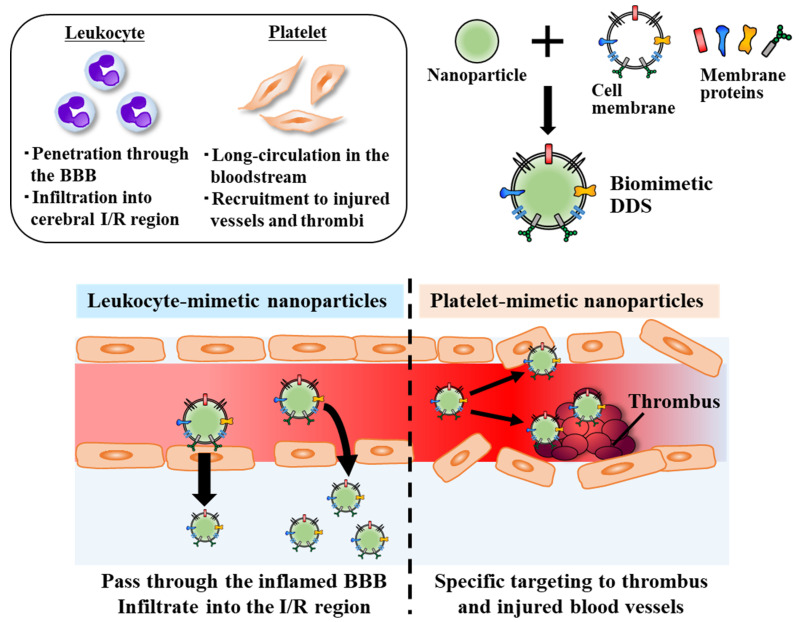
Biomimetic DDS prepared using circulating blood cells (leukocytes and platelets) for the treatment of ischemic stroke.

Blood cells circulating in our bodies have intrinsic properties that are advantageous for drug delivery applications [95]. In particular, certain types of cells exhibit the ability to pass through biological barriers, including the BBB, via membrane protein functions, while avoiding undesired immune responses. Research efforts focused on the preparation of biomimetic DDS and their applications for ischemic stroke therapy are reviewed below and highlighted in Figure 3.

Leukocytes, especially neutrophils (which are the most abundant leukocytes in the bloodstream), work as guardians to protect our bodies from infections and injuries. On the other hand, interactions between vascular endothelial cells and leukocytes play a crucial role in the pathological progression of inflammatory diseases, including cerebral I/R injury [96]. The development of biomimetic nanoparticles mimicking leukocytes has been investigated, as leukocytes can actively infiltrate the injured BBB, in addition to inflamed vessels, via membrane proteins. Parodi et al. prepared nanoporous silicon synthetic nanoparticles coated with plasma membranes collected from leukocytes, termed leuko-like vectors (LLVs) [97]. Leukocyte membrane-coating was achieved via chemical conjugation and electrostatic interactions between negatively-charged membranes and positively-charged synthetic nanoparticles. LLVs were found to exhibit leukocyte-like functions and demonstrated the ability to pass through the inflamed endothelial cell layer in vitro and in vivo. It is suggested that coating the LLVs with membrane proteins allowed for activation of signaling pathways of endothelial cells, to subsequently induce an increase in vascular permeability, which enabled penetration of inflamed vessels, similar to leukocytes [98].

Dong et al. prepared neutrophil membrane-derived nanovesicles using neutrophil-differentiated human promyelocytic leukemia HL-60 cells as cellular sources [99]. The nanovesicles were prepared by first breaking the cells by nitrogen cavitation to obtain nanovesicles, followed by nanovesicle purification via a series of centrifugation steps. Then, the therapeutic agent Resolvin D (RvD), which is derived from docosahexaenoic acid and exhibits a lipid structure, was incorporated into the lipid bilayer membranes of the nanovesicles. RvD was previously reported to alleviate inflammation by inhibition of endothelial cell activation, neutrophil infiltration, and cytokine release in other inflammatory disease models [100]. The RvD-modified nanovesicles showed specific targeting capabilities against inflamed cerebral endothelial cells in ischemic stroke model mice by inspiring interactions between cerebral endothelial cells and neutrophils. Moreover, treatment with RvD-modified nanovesicles significantly reduced brain damage by inhibition of intrinsic neutrophil recruitment in the I/R region [99]. Although BBB penetration by the nanovesicles was not mentioned in the report, the RvD-modified nanovesicles were suggested to be useful for the treatment of a variety of inflammatory diseases.

In another report, Feng et al. developed a neutrophil-like cell membrane-coated mesoporous Prussian blue nanozyme (MPBzyme@NCM) using cell membranes isolated from neutrophil-differentiated HL-60 cells [101]. The nanozyme had previously been chemically designed to possess ROS-scavenging and anti-inflammatory activities [102,103]. Coating the nanozyme with HL-60-derived neutrophil membranes was performed by extrusion with 100-nm polycarbonate membrane filters using an extruder. Similar to the report from Dong et al. [99], neutrophil-membrane coating of the nanozyme brought about specific targeting abilities to inflamed brain endothelial cells via the functions of the membrane proteins. Importantly, by mimicking neutrophil infiltration through the inflamed BBB, MPBzyme@NCM efficiently passed through the inflamed BBB and reached the brain parenchyma around the I/R region. After accumulation in the brain parenchyma, MPBzyme@NCM was subsequently taken up by the microglia and exerted antioxidative and anti-inflammatory effects, resulting in long-term therapeutic effects [101]. These findings indicate that coating the nanoparticle surface with leukocyte membranes allows for active targeting of the resultant nanoparticles to the inflamed endothelium. Nanoparticle delivery into the ischemic region via active passage through the inflamed BBB may thus be possible by capitalizing on the functions of leukocyte membranes.

As another example of biomimetic DDS utilizing circulating cells, platelet membrane-coating has also been investigated for the treatment of a variety of diseases. Platelets can circulate in the bloodstream for long periods (half-life: 30 h) and are involved in thrombus formation, in which activated platelets are recruited to the site of injured vessel walls and become one of the main components of the thrombi. Owing to the unique properties of platelets, nanoparticles coated with platelet membranes have also been applied for the treatment of cancer and inflammatory diseases (e.g., infections and atherosclerosis) [104,105,106]. Xu et al. developed a thrombin-responsive platelet membrane-coated nanoparticle system to achieve sequential, site-specific delivery of t-PA and the neuroprotectant ZL006 to the thrombus region and ischemic brain parenchyma [107]. The dextran derivative polymeric nanoparticles encapsulating ZL006 were coated with platelet membranes, followed by modification of the platelet membranes with the cell-penetrating peptide Tat. Then, t-PA was chemically conjugated onto the tip of the Tat peptides by introducing a thrombin cleavable amino acid sequence as a linker. As thrombin markedly accumulates in thrombus sites [108], the platelet-inspired long-circulating nanoparticles can rapidly release t-PA to the thrombi in a thrombin-triggered manner via degradation of the thrombin cleavable linker. After lysis of the thrombi by t-PA, the exposed Tat peptides conjugated to the platelet membranes interact with the injured BBB, resulting in Tat-mediated active penetration of the injured BBB and efficient ZL006 delivery into the ischemic brain. The authors concluded that platelet-mimetic and thrombin-responsive sequential drug delivery should offer a promising strategy for the treatment of ischemic stroke [107].

### 5.2. Development of Biomimetic DDS via Intermembrane Protein Transfer

The research findings described above suggest that the design of biomimetic DDS that mimic the properties of leukocytes and platelets could become an attractive strategy to overcome biological barriers and increase the utility of nanoparticle-mediated drug delivery for the treatment of ischemic stroke. On the other hand, issues with establishing standard procedures for the preparation of biomimetic nanoparticles have been reported, as complicated processes are required for isolation of cellular membrane/membrane proteins and their coating onto nanoparticles may lead to loss of protein activities and native structural orientations, as well as difficulty in regulating physicochemical properties [109]. Besides, some circulatory cells are difficult to harvest and handle while also maintaining their cellular integrity [110]. Therefore, an approach that easily allows for imparting the unique properties of circulatory cells onto nanoparticles will be useful for the development of biomimetic DDS.

It was previously reported that a variety of membrane proteins can be spontaneously transferred from cellular membranes to the liposomal bilayer through a phenomenon known as intermembrane protein transfer [111]. By employing this phenomenon, reconstitution of membrane proteins, with their functions retained, was performed onto artificial lipid membranes without intricate processes for protein purification, solubilization, and reconstitution. Huestis et al. previously demonstrated that an erythrocyte membrane protein (anion transport carrier band 3) retained its original activity and native orientation following transfer onto liposomal membranes [111]. Okumura et al. reported facile transfer of platelet membrane proteins onto liposomes with minimal contamination from soluble cytosolic proteins compared to conventional reconstitution methods [112]. Intermembrane protein transfer was reported to occur only by incubation of liposomal suspensions and cells, while its precise mechanism is still a matter of controversy. One proposed mechanism suggests that when a partial fusion of the external leaflet is transiently elicited through contact between cells (protein donor) and lipid bilayer membranes of liposomes (protein acceptor), donor membrane proteins spontaneously transfer to the acceptor membranes [111]. The intermembrane protein transfer can be applied to protein reconstitution onto not only liposomes but also erythrocyte ghosts. Kogure et al. constructed erythrocyte ghosts with membrane fusible capabilities by transferring viral glycoprotein hemagglutinin (HA) from HA-overexpressed cells [113]. In addition, the membrane-fusible erythrocyte ghosts were found to achieve intracellular delivery of encapsulated proteins with retained activities [114]. With regard to the therapeutic applications of such systems, Shibata et al. prepared tumor-antigen-transferred liposomes via reconstitution of membrane proteins derived from target cancer cells [115]. Subcutaneous injection of the liposomes into tumor-bearing mice effectively activated the immune response and suppressed tumor growth. These findings indicate that intermembrane protein transfer may be a convenient method to develop biomimetic DDS through protein transfer from isolated circulatory cells or cultured cells without the need for complicated reconstitution steps.

By employing intermembrane protein transfer, Fukuta et al. developed liposomes modified with leukocyte membrane proteins, which are referred to as leukocyte-mimetic liposomes (LM-Lipo) [116,117,118]. Among the various mechanisms of leukocyte penetration through the inflamed endothelium, the authors focused on the interaction of two leukocyte membrane proteins, namely lymphocyte function-associated antigen-1 (LFA-1; CD11a) and macrophage antigen-1 (Mac-1; CD11b), with intercellular adhesion molecule (ICAM)-1 expressed on the inflamed endothelium [119]. The binding of both CD11a and CD11b to ICAM-1 was reported to induce activation of the ICAM-1 signaling pathway in endothelial cells and increase vascular permeability, resulting in leukocyte migration into the inflamed region [120]. To mimic this biological response, LM-Lipo was prepared via intermembrane protein transfer using neutrophil-differentiated HL-60 cells as donor cells, on which both CD11a and CD11b were expressed [121]. LM-Lipo exhibited a higher affinity to inflamed human umbilical vein endothelial cells (HUVECs) compared with non-inflamed cells. Moreover, treatment with LM-Lipo induced a reduction in vascular-endothelial cadherin expression, which contributed to the passage of the liposomes through the inflamed HUVEC layer, similar to leukocytes [117]. These results demonstrated that leukocyte membrane proteins transferred onto liposomes retained their activities and exerted leukocyte-mimetic functions. The utility of LM-Lipo as a drug carrier was also demonstrated using a tumor spheroid model, in which LM-Lipo encapsulating an anti-cancer drug was shown to efficiently infiltrate the spheroid and suppress its growth compared with plain liposomes [118]. Based on these findings, intermembrane protein transfer offers a promising alternative approach to the development of biomimetic DDS, in addition to other methods such as the abovementioned cell-membrane coating approach.

## 6. Concluding Remarks and Future Perspectives

In this article, recent efforts to investigate nanoparticle-based DDS, especially liposomes, in the interest of developing therapeutic agents for the treatment of ischemic stroke have been reviewed. Liposomes and other synthetic nanoparticles can carry therapeutic drugs that exhibit a number of different physical properties, such as one of a range of molecular sizes (from small to macromolecular) and hydrophilicity/hydrophobicity, by encapsulation or surface modification, which brings about efficient delivery of the drugs to diseased areas and an increase in their therapeutic effects. Accordingly, nano-sized DDS drugs exhibit remarkable therapeutic effects and ameliorate brain injury induced by ischemic stroke and subsequent I/R injury. Moreover, surface modification of nanoparticles with certain ligands against targeting molecules specifically expressed in the lesioned sites can lead to a further increase in nanoparticle delivery efficiency to the ischemic stroke region. Further, combination therapy comprised of DDS drugs with t-PA, a gold-standard thrombolytic agent, offers the potential to extend the narrow TTW of t-PA, while reducing the risk of adverse side effects following thrombolysis. As disruption of the BBB following an ischemic stroke has been reported to take place in human patients as well as animal models [122,123], therapeutic strategies using nanoparticulate DDS may offer great potential for application to ischemic stroke therapy in humans. In recent years, the application of biomimetic DDS exhibiting the unique properties of circulating cells, such as leukocytes and platelets, has been investigated to achieve more reliable drug delivery than conventional ligand-mediated active targeting strategies. As those blood-circulating cells can cross the BBB by means of membrane protein functions, imparting such functions onto nanoparticles is considered a hopeful approach to overcoming limitations associated with the BBB. Our research efforts to easily prepare biomimetic liposomes by a facile method, namely intermembrane protein transfer, were also highlighted. To realize the clinical application of the abovementioned nanoparticle DDS for delivery of cerebroprotective agents, numerous issues remain to be addressed, such as precise evaluation of the pharmacokinetics and safety/tolerability, along with the establishment of industrialization processes for mass production, among others. However, it is suggested that the application of nanoparticle DDS may be a useful strategy for the development of novel therapeutic agents for ischemic stroke.

## Figures and Tables

**Table 1:** List of liposomal neuroprotective agents for the treatment of ischemic stroke (described in this review).

Drug	Main Mechanism of Action	Effectiveness	References
Asialo-erythropoietin (AEPO)	Anti-apoptotic effect through binding to EPO receptors	Reduced damaged brain volume via anti-apoptosis and improved motor functional deficits	[22,24]
Fasudil	Inhibition of Rho-kinase activation	Suppressed neutrophil infiltration and ameliorated ischemic brain damage	[25,28]
FK506 (Tacrolimus)	Anti-inflammatory effect via calcineurin inhibition	Ameliorated ischemic brain damage by anti-inflammatory and anti-apoptotic effects from acute to subacute phases	[31,32]
Cyclosporin A	Anti-inflammatory effect via calcineurin inhibition	Suppressed inflammation through inhibition of release of inflammatory cytokines from immune cells and neurons	[34]
ZL006	Prevention of glutamate-induced excitotoxicity through inhibition of NMDAR signaling	Suppressed brain damage induced by cerebral I/R injury and improved motor functions	[36,42]
Ifenprodil	Inhibition of NMDAR-mediated glutamate excitotoxicity	Ameliorated ROS-mediated BBB damage and suppressed brain damage	[37]
Baicalin	Antioxidative effects through ROS scavenging	Improved biodistribution and brain accumulation of baicalin (No mention about therapeutic effect)	[38]
Edaravone	Antioxidative effect through ROS scavenging	Reduced neuronal cell damage and suppressed brain damage	[39]
Simvastatin	Pleiotropic effects of 3-hydroxy-3-methylglutaryl coenzyme A reductase inhibition	Improved biodistribution and brain accumulation of simvastatin	[40]
Citicoline	Membrane repair and regeneration Alleviation of fatty acid-induced toxicity	Suppressed ischemic brain damage and edema	[41,43]

**Table 2:** List of ligands targeted to the ischemic stroke region, as described in this review.

Ligand	Target Molecule	References
Anti-HSP72 antibody	HSP72	[43]
T7 peptide (HAIYPRH)	Transferrin receptor (TfR) (Expressed on cerebral endothelial cells)	[44,45]
Stroke-homing peptide (SHp; CLEVSRKNC)	Unknown (Possible target: activated glutamate receptors)	[42,47]
AEPO	EPO receptors (Upregulated on cerebral endothelial cells and neurons after ischemic stroke)	[24]
cRGD (Arg-Gly-Asp-_D_-Tyr-Lys)	Integrin αvβ1, αvβ3 (Expressed on inflamed endothelial cells and leukocytes) Glycoprotein IIb-IIIa (Expressed on activated platelets in thrombus)	[39,50,53,56]
Chlorotoxin	MMP-2 (Upregulated in the ischemic stroke region)	[57,58]

**Table 3:** List of combination therapies and nanoparticle applications for t-PA delivery, as described in Section 3.

Application	Outcome	References
PEGylated liposomes encapsulating fasudil plus t-PA	Suppression of t-PA-induced BBB damage and MMP-2, 9 activation, and amelioration of brain damage and motor functional deficits	[71,72]
PEGylated liposomes	Encapsulation of t-PA in liposomes Prolongation of blood circulation period of t-PA by avoiding enzymatic degradation	[75]
Gold nanoparticles	Chemical conjugation of t-PA onto the nanoparticles Prolongation of blood circulation time	[76]
4-amino-TEMPO-conjugated redox nanoparticles	Extension of half-life of t-PA with enzymatic activities Prevention of oxidative stress and ameliorated cerebral I/R injury	[77]
